# Overcoming Immune Barriers in Allogeneic CAR-NK Therapy: From Multiplex Gene Editing to AI-Driven Precision Design

**DOI:** 10.3390/biom15070935

**Published:** 2025-06-26

**Authors:** Hyunyoung Kim

**Affiliations:** 1 Division of Intractable Disease Research, Department of Chronic Convergence Research, Korea National Institute of Health, Cheongju 28160, Republic of Korea; inbiology@korea.kr; 2 Korea National Stem Cell Bank, Cheongju 28160, Republic of Korea

**Keywords:** CAR-NK cells, gene editing, immune evasion, allogeneic cell therapy, iPSC-derived NK cells, B2M knockout, HLA-E overexpression, CD47, artificial intelligence, TIGIT blockade

## Abstract

Chimeric antigen receptor (CAR)-engineered natural killer (NK) cells are a promising platform for off-the-shelf immunotherapy due to their safety advantages over CAR-T cells, including lower risk of graft-versus-host disease, cytokine release syndrome, and neurotoxicity. However, their persistence and efficacy are limited by immunological challenges such as host T-cell-mediated rejection, NK cell fratricide, and macrophage-mediated clearance. This review summarizes gene editing strategies to overcome these barriers, including β2-microglobulin (B2M) knockout and HLA-E overexpression to evade T and NK cell attacks, CD47 overexpression to inhibit phagocytosis, and TIGIT deletion to enhance cytotoxicity. In addition, we discuss functional enhancements such as IL-15 pathway activation, KIR modulation, and transcriptional reprogramming (e.g., FOXO1 knockout) to improve persistence and antitumor activity. We also highlight the role of induced pluripotent stem cell (iPSC)-derived NK platforms, enabling standardized, scalable, and multiplex gene-edited products. Finally, we explore artificial intelligence (AI) applications in immunogenomic profiling and predictive editing to tailor NK cell therapies to patient-specific HLA/KIR/SIRPα contexts. By integrating immune evasion, functional reinforcement, and computational design, we propose a unified roadmap for next-generation CAR-NK development, supporting durable and broadly applicable cell-based therapies.

## 1. Introduction

Natural killer (NK) cells are cytotoxic lymphocytes that play a central role in innate immunity by eliminating virally infected and transformed cells without prior sensitization. Unlike T cells, NK cells can recognize stressed or altered cells through a balance of activating and inhibitory signals rather than antigen-specific T-cell receptors, offering a rapid and MHC-unrestricted cytolytic response [1].

The clinical success of chimeric antigen receptor T (CAR-T)-cell therapy has revolutionized the treatment of hematological malignancies, particularly B-cell-derived cancers. However, widespread application remains limited due to cytokine release syndrome (CRS), neurotoxicity, and high manufacturing cost and time. These challenges have accelerated interest in alternative immune cell types such as NK cells, which exhibit lower risk of CRS and graft-versus-host disease (GVHD) while retaining potent antitumor functions [2].

Compared to autologous approaches, allogeneic NK cell therapy offers the potential to develop “off-the-shelf” immune cell products that can be standardized, cryopreserved, and made rapidly available for multiple patients. Several clinical trials using cord blood-derived or iPSC-derived NK cells have reported encouraging results in hematological malignancies and are expanding into solid tumors [3]. However, the large-scale manufacturing of NK cells remains challenging due to limited expansion capacity, variable transduction efficiencies, and the need for robust GMP-compliant production platforms that ensure consistency and clinical-grade quality.

Despite these advantages, the translation of allogeneic NK cell therapies to the clinic faces a central bottleneck: immune rejection by the host. Infused NK cells are rapidly eliminated by host immune cells, including CD8^+^ T cells, host NK cells recognizing “missing self,” and macrophages mediating antibody-independent phagocytosis. Moreover, NK cells themselves can engage in fratricide or become dysfunctional due to inhibitory signaling from the tumor microenvironment [4]

Gene editing technologies, particularly CRISPR/Cas9 and TALEN, have enabled precise manipulation of NK cells’ immunogenicity, persistence, and function. A variety of editing strategies have been explored to enhance compatibility with host immunity: (1) modulating HLA class I expression via B2M or HLA-E to avoid T-cell- and NK-cell-mediated clearance, (2) overexpressing CD47 to suppress macrophage-mediated elimination, and (3) disrupting inhibitory checkpoint pathways such as PD-L1, TIGIT, or CISH to improve cytotoxicity and tumor infiltration [5].

In this review, we examine the immune barriers limiting the persistence of allogeneic NK cells, critically evaluate gene editing strategies to overcome these barriers, and propose a forward-looking framework that incorporates AI-guided design and bioinformatics-based personalization. Our goal is to provide a comprehensive yet strategic analysis that moves beyond cataloging individual targets, aiming instead to map the field’s trajectory toward durable, safe, and universal NK cell therapies.

## 2. CAR Design Considerations in NK Cell Therapy

Chimeric antigen receptor (CAR) design is a critical determinant of therapeutic efficacy, safety, and persistence in NK-cell-based immunotherapy [6]. While foundational CAR components are shared with T-cell applications, several NK-specific design features have emerged.

Target antigen selection remains pivotal. In hematological malignancies, CD19, CD22, and BCMA are widely used, while solid tumors require more selective targets to avoid off-tumor effects. Novel approaches such as dual CARs and SynNotch receptors aim to increase specificity and reduce escape variants [7]. The choice of co-stimulatory domains significantly impacts NK cell signaling. Domains like 2B4, DAP10, and NKG2D have shown superior synergy with NK physiology compared to T-cell-centric domains such as CD28 or 4-1BB. These NK-preferred domains enhance IFN-γ production and degranulation while reducing exhaustion [8]. Vector platforms also affect CAR performance. Lentiviral and retroviral vectors offer stable integration but carry insertional mutagenesis risks. Transposon systems (e.g., Sleeping Beauty, PiggyBac) and mRNA electroporation are gaining attention for non-viral, footprint-free engineering. In particular, mRNA-based transient expression may be preferred in early-phase trials due to enhanced safety. Recent developments in non-viral gene delivery using Cas9 ribonucleoproteins have also enabled efficient and precise CAR integration in primary NK cells, with minimized off-target effects [9].

Altogether, optimal CAR design for NK cells requires antigen selectivity, signal tuning, and a delivery platform tailored to the intended therapeutic context.

## 3. Immunological Barriers in Allogeneic NK Cell Therapy

While allogeneic NK cell therapy offers the promise of off-the-shelf immunotherapy, its efficacy is undermined by complex host immune responses that target infused cells. These barriers, mediated by cytotoxic T cells, NK cells, macrophages, and the tumor microenvironment, are not isolated challenges but interconnected forces that evolve dynamically after cell infusion. Addressing these immune pressures requires a holistic understanding of how editing strategies interact with host immunity and tumor context.

### 3.1. CAR Engineering and Combinatorial Strategies to Overcome TME-Induced Suppression

The tumor microenvironment (TME) imposes major barriers to CAR-NK cells’ efficacy, including immunosuppressive cytokines (e.g., TGF-β, IL-10), metabolic constraints such as hypoxia and adenosine accumulation, and immune checkpoints like PD-L1. Several CAR engineering strategies have been developed to counteract these suppressive factors.

One promising approach involves expressing dominant-negative receptors that abrogate TGF-β signaling. For instance, CAR-NK cells engineered to express dominant-negative TGF-β receptor II (dnTGF-βRII) have demonstrated enhanced resistance to TME-mediated suppression [10,11].

Another strategy includes the use of switch receptors, such as PD1:CD28 chimeras, which convert inhibitory signals into activating ones. This design has shown efficacy in preclinical models by improving the cytotoxicity and persistence of engineered cells [12,13].

Additionally, co-expression of cytokines like IL-15 or membrane-bound IL-12, along with hypoxia-responsive CAR elements, is being explored to sustain NK cell activity in metabolically stressed TME conditions [14,15]. The incorporation of chemokine receptors such as CXCR4 also improves tumor-homing potential [16]. Combinatorial strategies involving checkpoint inhibitors (e.g., anti-PD-1 antibodies), STING agonists, and oncolytic viruses have shown synergistic potential in modulating the TME and enhancing CAR-NK efficacy.

### 3.2. Host T-Cell-Mediated Rejection and the Challenges of HLA Modulation

Host CD8^+^ T cells play a central role in eliminating allogeneic NK cells through the recognition of mismatched HLA class I molecules, particularly HLA-A and HLA-B. To reduce this immunogenicity, gene editing strategies commonly target β2-microglobulin (B2M) or remove selected HLA alleles, thereby limiting antigen presentation to host T cells [17].

However, complete ablation of HLA class I expression disrupts interactions with host inhibitory NK receptors, potentially triggering NK-cell-mediated lysis through the “missing-self” response. To mitigate this, selective retention of HLA-C—recognized by inhibitory KIRs—is increasingly favored. However, given the diversity of KIR-HLA interactions across individuals, this strategy’s success may depend on patient-specific immune profiling. What appears to be a universally “stealth” cell product may still provoke immune recognition in KIR-mismatched recipients [18].

### 3.3. NK-Cell-Mediated Clearance: The Complexity of the “Missing Self”

Host NK cells patrol for cells lacking self-HLA signals and rapidly eliminate those that fail to engage inhibitory receptors such as KIR or NKG2A. Overexpression of non-classical HLA-E, which binds to NKG2A, has been proposed to suppress this immune activation [19]. While effective in preclinical models, this approach is not universally reliable. Single-cell transcriptomics data from cancer patients show that tumor-infiltrating NK cells often have reduced NKG2A expression and elevated activating receptors like NKG2C and KIR2DS1. In such contexts, HLA-E expression may not only fail to protect infused NK cells but could paradoxically enhance their visibility to the immune system.

This observation underscores a broader issue in universal cell design: the efficacy of immune evasion strategies is conditioned by the immune architecture of the recipient. A single strategy, such as HLA-E overexpression, may be insufficient—or even counterproductive—without matching to the NK receptor phenotype of the host.

### 3.4. Phagocyte-Mediated Clearance and the Duality of CD47

In addition to lymphocyte-mediated rejection, macrophages contribute to the early clearance of infused NK cells, particularly in reticuloendothelial tissues such as the liver and spleen. CD47, a transmembrane protein that binds SIRPα on macrophages, delivers an inhibitory signal that prevents phagocytosis. Overexpression of CD47 on therapeutic NK cells has been shown to reduce macrophage clearance and prolong circulation time in vivo [20].

However, this strategy must be applied with caution. CD47 is a well-characterized immune evasion molecule in tumor biology, and its overexpression in therapeutic cells raises concerns about off-target effects, including interference with dendritic-cell-mediated antigen presentation and the inadvertent protection of residual tumor cells. Moreover, macrophage activity varies by tissue type and disease state, suggesting that a one-size-fits-all CD47 strategy may not be appropriate. Conditional or tissue-specific expression systems could help balance persistence and safety in future designs.

### 3.5. Suppression Within the Tumor Microenvironment

Even when NK cells evade host immune rejection, their functional longevity remains at risk within the suppressive milieu of the tumor microenvironment (TME). TGF-β, IL-10, hypoxia, and metabolic constraints all contribute to NK cell dysfunction post-infiltration. Additionally, tumor cells upregulate immune checkpoint ligands such as PD-L1 and CD155, while recruiting regulatory T cells and myeloid-derived suppressor cells that inhibit NK cells’ activation and cytotoxicity [21].

These environmental pressures drive the upregulation of inhibitory receptors like TIGIT, TIM-3, and LAG-3, which are often co-expressed with exhaustion markers in NK cells recovered from solid tumors [22]. While many gene editing strategies focus on early immune evasion, sustained therapeutic activity may require parallel editing of intrinsic regulatory genes that enhance resistance to TME-induced dysfunction.

Taken together, these immunological barriers illustrate that engineering persistence in NK cell therapy is not a matter of evading one immune cell type. Instead, it requires layered strategies that account for immune heterogeneity, tissue context, and dynamic suppression mechanisms. Future platforms must move beyond isolated target editing toward systems-level designs informed by patient- and tumor-specific immune profiles.

**Figure 1:**
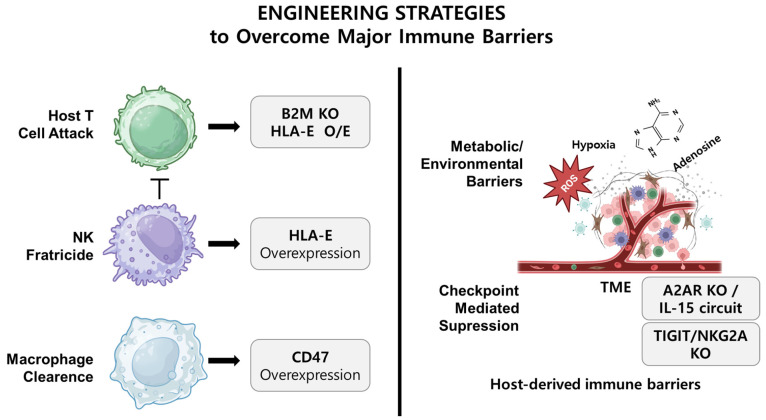
Engineering strategies to overcome host- and TME-derived immune barriers in allogeneic CAR-NK cell therapy: This schematic illustrates the major immunological and microenvironmental barriers that impair the efficacy of allogeneic CAR-NK cell therapy, along with representative gene editing strategies designed to overcome each challenge. Created in https://BioRender.com.

To address these multifaceted immune barriers, several targeted gene editing strategies have been developed. Figure 1 provides an updated schematic representation of the key immune barriers faced by allogeneic CAR-NK cells and their corresponding gene editing solutions, including both host-derived immune recognition and tumor microenvironmental suppression.

## 4. Gene Editing Strategies to Enable Immune Evasion

To systematically overcome these immune barriers, diverse engineering strategies have been developed to enhance the function and persistence of CAR-NK cells. Advances in genome editing have enabled precise modification of NK cells’ immunogenicity, allowing developers to engineer allogeneic NK cells that can avoid recognition and elimination by host immune cells. These strategies, targeting distinct immune rejection pathways, are designed to suppress T-cell- and NK-cell-mediated clearance, reduce macrophage phagocytosis, and improve immunological stealth. This section focuses on the editing strategies that are most widely employed to facilitate immune evasion in allogeneic NK cell therapies, with an emphasis on their mechanisms, current preclinical/clinical applications, and potential combinatorial use.

### 4.1. Modulating HLA Class I Expression: From Deletion to Precision Retention

To reduce recognition by host CD8^+^ T cells, many groups have employed B2M knockout to eliminate surface expression of HLA-A, HLA-B, and HLA-C molecules. This strategy effectively suppresses antigen presentation and can mitigate alloreactive T-cell activation [17].

However, complete HLA class I loss triggers missing-self responses from host NK cells. To balance these effects, several groups now adopt a more refined strategy: deletion of HLA-A and -B alleles while retaining HLA-C expression. Since HLA-C engages inhibitory KIRs on NK cells, this selective retention preserves inhibitory signaling and avoids NK-mediated clearance. TALEN-based approaches and CRISPR/Cas9 with allele-specific guides have been applied to enable this selective editing [23].

More recently, fusion constructs such as B2M–HLA-E or B2M–HLA-G have been designed to replace classical HLA molecules with immunomodulatory non-classical ones. These constructs allow surface expression in the absence of B2M, engaging NKG2A or KIR2DL4 receptors on host NK and T cells to transmit inhibitory signals [17]. While promising, such approaches must be evaluated for their capacity to modulate both innate and adaptive immune responses without compromising tumor-targeting efficacy.

### 4.2. Overexpression of CD47 to Evade Phagocytosis

Macrophage-mediated clearance can significantly shorten the persistence of infused NK cells, especially in organs such as the liver and spleen. To address this, CD47 has been overexpressed in engineered NK cells to inhibit phagocytosis via interaction with SIRPα on macrophages [24,25].

Several preclinical models demonstrate that CD47-overexpressing NK cells show prolonged circulation and improved tumor localization. For example, one study reported a threefold increase in the in vivo persistence of CD47-engineered NK cells compared to unmodified controls [24,26].

Despite these benefits, the risk of immunosuppressive overlap with tumor immune evasion remains. While CD47 overexpression prolongs NK cells’ persistence, its potential to mimic tumor immune evasion should not be overlooked. Conditional or inducible CD47 expression may offer a safer alternative, especially in solid tumor settings where macrophage function is critical for immune priming. Incorporating tissue-specific promoters or switchable expression systems could balance persistence with immunological safety. Therefore, approaches are under development to regulate CD47 expression temporally or spatially, such as by using inducible promoters, tissue-restricted elements, or microRNA-based repression in non-target tissues.

### 4.3. Engineering HLA-E to Suppress Host NK Activation

Another frequently used immune evasion strategy is the overexpression of HLA-E, a non-classical HLA molecule that binds to NKG2A/CD94-inhibitory receptors. HLA-E can restore NK-inhibitory signaling in settings where classical HLA molecules are absent, particularly after B2M knockout [19].

Gene editing platforms have integrated HLA-E cDNA into NK cell genomes using CRISPR/Cas9-mediated knock-in or lentiviral delivery. Some iPSC-derived NK products now routinely include HLA-E overexpression to suppress alloreactive NK cell clearance [27].

However, the effectiveness of this approach is highly dependent on the host NK cell phenotype. In recipients where NKG2A is not dominantly expressed, HLA-E may fail to suppress NK activity or may even activate certain NK subsets (e.g., NKG2C+ or KIR2DS+). Thus, HLA-E-based evasion is best viewed as context-specific, potentially requiring pre-infusion profiling of the recipient’s NK cell repertoire [28,29].

### 4.4. KIR Editing: Balancing Autonomy and Tolerance

Modulating KIR expression offers a complementary strategy for NK cell evasion. By deleting inhibitory KIRs such as KIR2DL1 or KIR2DL3, NK cells can become resistant to suppression by tumor-expressed HLA ligands, enhancing their cytotoxic potential [30,31].

Conversely, engineering NK cells to overexpress activating KIRs such as KIR2DS1 can boost their functional activity in tumors that retain corresponding HLA-C ligands. Several studies have demonstrated that KIR mismatch models—in which donor NK cells express activating KIRs without matching inhibitory HLA ligands—can promote enhanced cytotoxicity in acute myeloid leukemia and other malignancies [32,33].

The challenge is maintaining sufficient self-tolerance to avoid off-tumor effects, particularly in non-hematological tissues. Therefore, selective editing of KIR subsets, rather than their complete deletion, is being explored.

To provide a systematic comparison of the major gene editing strategies targeting immune rejection pathways in allogeneic NK cell therapy, we summarize the key molecular targets, mechanisms, and known limitations in Table 1.

## 5. Functional Enhancement via Intrinsic Regulator Editing

Beyond extrinsic immune evasion, the intrinsic regulatory machinery of NK cells can be genetically tuned to further boost cytotoxicity and survival. While gene editing for immune evasion has advanced the feasibility of allogeneic NK cell therapy, avoiding clearance is only the first step. To achieve durable antitumor effects, engineered NK cells must persist in vivo, remain cytotoxic within immunosuppressive environments, and resist functional exhaustion. These requirements are especially critical in solid tumors, where the tumor microenvironment exerts sustained metabolic and immunological stress. Increasingly, attention is shifting from external immune escape to internal resilience—achieved by editing key regulators of NK cell signaling, metabolism, and memory-like function.

### 5.1. Targeting CISH to Unlock IL-15 Responsiveness

Among intrinsic inhibitors, the cytokine-inducible SH2-containing protein (CISH) has emerged as a potent intracellular brake on NK cell activity. CISH negatively regulates IL-15 signaling, which is central to NK cells’ homeostasis, activation, and persistence. CRISPR-mediated deletion of CISH has been shown to dramatically increase NK cells’ proliferation, cytotoxic granule release, and in vivo persistence following adoptive transfer [41].

Notably, this editing strategy complements external cytokine support systems, such as IL-15-armored CAR-NK cells, by enhancing downstream sensitivity. However, uncontrolled IL-15 signaling may pose a risk of NK cell overactivation and associated toxicities, including bystander killing or tissue infiltration. These concerns highlight the need for fine-tuning rather than full deregulation, possibly through inducible CISH repression or partial editing approaches [42,43].

From a translational standpoint, CISH editing represents a promising lever for improving NK cell function in both hematological and solid tumors—especially in patients where exogenous cytokine support is limited or toxic.

### 5.2. Disrupting Transcriptional Exhaustion via FOXO1

Another intrinsic barrier to sustained NK cell activity is transcriptional exhaustion. The forkhead transcription factor FOXO1 has been identified as a negative regulator of NK cell effector differentiation and memory-like conversion. Loss of FOXO1 leads to increased expression of T-bet and Eomes, two transcription factors associated with robust cytotoxic function and metabolic resilience [44,45].

While the majority of FOXO1 studies are derived from murine models, the mechanistic parallels in human NK cells suggest a conserved pathway. However, complete deletion may also impair cell survival or homeostasis. An alternative approach may be to combine transient FOXO1 repression with metabolic reprogramming, thereby creating a window of enhanced activation without compromising long-term viability [46,47].

Crucially, FOXO1-targeted strategies may be especially beneficial in hostile microenvironments such as hypoxic solid tumors, where metabolic fitness and epigenetic flexibility are required for sustained infiltration and function [48].

### 5.3. Enhancing Infiltration and Response via IL-1R8 Knockout

IL-1 receptor 8 (IL-1R8), also known as SIGIRR, is a negative regulator of IL-1 family cytokine signaling and has been implicated in limiting NK cell activation and tumor infiltration. Genetic deletion of IL-1R8 leads to increased IFN-γ secretion, enhanced chemokine receptor expression (e.g., CXCR3, CCR5), and superior infiltration of NK cells into tumor sites [40,49].

What distinguishes IL-1R8 from other targets is its dual role in activation and trafficking, making it a compelling candidate for improving solid tumor responses. Moreover, IL-1R8 deletion does not rely on antigen-specific activation, suggesting that this modification could enhance both innate and CAR-engineered NK cell products.

However, the risk of overactivation in inflamed tissues—such as in autoimmune-prone patients—must be addressed. Future studies should explore whether IL-1R8 knockout synergizes with other edits, such as PD-L1 suppression or TIGIT deletion, to form a robust resistance program against tumor-induced suppression [50].

### 5.4. Beyond Checkpoints: Toward Durable NK Cell States

While individual genes such as CISH, FOXO1, and IL-1R8 offer discrete benefits, their true power may lie in combination. Emerging evidence suggests that editing multiple intrinsic regulators can induce a memory-like or trained immunity phenotype in NK cells, characterized by prolonged cytokine responsiveness, mitochondrial fitness, and stable effector programs [41,51].

Recent AI-based tools, such as NetMHCpan for epitope binding prediction [52] and MHCflurry 2.0 for immunogenicity modeling [53], have been incorporated into CAR design strategies to optimize antigen selectivity, particularly in the context of tumor heterogeneity and immune escape variants.

This concept represents a paradigm shift: rather than reactively restoring NK cell function after suppression, the goal becomes to precondition NK cells with durable antitumor potential before infusion. Such editing strategies may be most impactful when integrated with epigenetic modifiers or engineered metabolic switches, further enhancing their adaptability to dynamic tumor environments. Beyond immune evasion, intrinsic reprogramming of NK cells’ biology is increasingly recognized as essential for sustained antitumor activity. Table 2 outlines key internal regulatory genes that have been targeted to enhance NK cells’ persistence, metabolism, and cytotoxic function.

## 6. Emerging Integrative Strategies and Platforms

Emerging approaches are now integrating multiple engineering layers to tackle immune escape from several angles simultaneously. The rapid evolution of gene editing has produced an impressive array of individual strategies to enhance the efficacy and persistence of allogeneic NK cells. However, their true therapeutic potential will likely depend on how these strategies are combined, adapted to diverse tumor contexts, and embedded within scalable manufacturing platforms. The next frontier in NK cell engineering lies in building integrated systems that can navigate not only immune rejection but also manufacturing, safety, and patient variability [55,56].

### 6.1. Multiplex Gene Editing: Opportunities and Trade-Offs

The layered immune challenges faced by allogeneic NK cells—ranging from host T-cell rejection to tumor-induced exhaustion—have fueled interest in multiplex gene editing approaches that can address multiple barriers simultaneously. For instance, some preclinical models have combined B2M knockout with HLA-E overexpression and CD47 upregulation, resulting in enhanced in vivo persistence and reduced phagocytic clearance [57]. This strategy has been exemplified in next-generation iPSC-derived CAR-NK cell products that incorporate multiple edits to enable immune evasion and functional persistence.

However, increasing the number of edits raises significant risks. Recent studies have reported that complex edits involving three or more loci can introduce chromosomal rearrangements, large deletions, or translocations—some of which may not be detectable by conventional QC assays [58,59,60].

Multiplex editing should be guided not only by feasibility but by functional necessity. Strategies that simply layer immune evasion genes without modeling their interactive effects may inadvertently generate products with antagonistic signaling or unpredictable immune profiles. Prioritizing edits based on recipient immune profiling and tumor microenvironment context will likely yield more robust and clinically relevant outcomes.

In response, several groups are now adopting safer, sequential editing strategies with orthogonal Cas systems or base editors. There is also growing interest in using single-insertion cassettes that encode multiple immunoregulatory proteins under tissue-specific or inducible promoters, thereby reducing the need for multiple genomic cuts.

Critically, multiplex design must consider functional antagonism among targets. For example, combining PD-L1 overexpression (to evade T cells) with TIGIT knockout (to boost NK activation) may lead to paradoxical immunomodulation. Rational design must therefore be guided by context-specific modeling and in vivo validation [61]. Several of these editing strategies have progressed to clinical evaluation, often in combination with CAR insertion or cytokine support. Table 3 summarizes current clinical trials employing gene-edited allogeneic NK cell therapies, highlighting editing platforms, targets, and clinical indications.

To assess the translational relevance of these multiplex strategies, recent clinical and preclinical studies provide valuable insight into their efficacy and safety profiles. A representative example is the Phase 1 trial NCT04555811, conducted by Fate Therapeutics, which evaluated FT538—an iPSC-derived CAR-NK cell product incorporating B2M knockout, CD38 knockout, and HLA-E overexpression. In patients with relapsed or refractory B-cell lymphoma, the trial reported an objective response rate (ORR) of 38%, and notably, no cases of cytokine release syndrome (CRS), neurotoxicity, or GVHD were observed. Mild, transient cytopenias and fevers were reported in less than 30% of patients [62].

In contrast, preclinical work using base-edited CAR-NK cells with four simultaneous edits (B2M, CD47, TIGIT, HLA-E) reported systemic toxicity in 3 of 10 animals, illustrating that overengineering may compromise safety [43]. These findings emphasize the need for a balanced, context-aware approach in multiplex CAR-NK design.

### 6.2. Engineering Against the Tumor Microenvironment

Among the most formidable barriers, the tumor microenvironment (TME) imposes complex metabolic and signaling challenges that demand specialized engineering solutions. The TME imposes profound immunosuppressive pressure on CAR-NK cells, particularly within solid tumors. Hypoxia, nutrient scarcity, and immunoregulatory signals such as adenosine and TGF-β collectively impair CAR-NK persistence and function [63]. Overcoming these TME-induced barriers is thus essential for maximizing therapeutic efficacy in non-hematological malignancies.

Recent advances in synthetic biology have enabled CAR-NK cells to resist hypoxic and metabolically hostile environments. Liu et al. described genetic circuits that enhance mitochondrial oxidative metabolism and upregulate glycolytic flux to sustain cytotoxicity under oxygen-poor conditions [63]. These modifications support NK survival while maintaining perforin and IFN-γ release in hypoxic tumor regions.

To counteract immune checkpoint-mediated suppression in the TME, NK-specific regulators such as TIGIT and NKG2A have been deleted using multiplex CRISPR systems. Cui et al. demonstrated that such deletions, coupled with IL-15 receptor engineering, enhance antitumor responses even in checkpoint-rich tumor sites. Furthermore, adenosine pathway suppression through A2AR knockout or CD73 inhibition has been shown to restore CAR-NK effector functions [64].

These insights collectively suggest that rational TME-focused CAR-NK engineering can substantially expand the therapeutic reach of allogeneic NK therapies, particularly in solid tumors. As summarized by Zhou et al., overcoming TME suppression remains one of the defining challenges in the next generation of cell-based immunotherapies [65].

These immunosuppressive pathways form only part of the tumor’s defense architecture. In parallel, the structural composition of the TME, particularly the extracellular matrix, poses additional physical obstacles that hinder immune cell infiltration.

The therapeutic efficacy of CAR-NK cells against solid tumors is critically influenced by their ability to infiltrate the tumor mass, which is often hindered by a dense extracellular matrix (ECM), collectively termed the oncomatrix. This fibrotic stroma, composed of collagen, hyaluronan, and matrix-bound proteins secreted by cancer-associated fibroblasts (CAFs), acts as a physical and biochemical barrier that restricts NK cell migration and immunological synapse formation [66]. To overcome this, strategies such as the co-expression of ECM-degrading enzymes (e.g., heparanase), use of matrix-targeted CAR constructs, and modulation of integrin or chemokine signaling pathways are being actively explored [67]. Additionally, modulation of tumor stiffness and normalization of stromal components are emerging as combinatorial approaches to improve immune cell access. Incorporating these insights into CAR-NK design is essential for enhancing therapeutic performance in the solid tumor context.

### 6.3. iPSC-Based Platforms for Programmable NK Cell Design

Induced pluripotent stem cells (iPSCs) provide a renewable, genetically stable source for NK cell production and multiplex engineering. Unlike peripheral blood- or cord blood-derived NK cells, iPSCs can be clonally edited at the pluripotent stage, ensuring homogeneity and traceability. Several companies have successfully produced iPSC-derived NK cells with integrated CARs, IL-15 transgenes, and immune evasion modules [56,62].

This model enables “design-then-expand” workflows, in contrast to conventional NK therapies that rely on the expansion of heterogeneous populations. Furthermore, the iPSC platform facilitates banking of master lines, quality-controlled at both the genome and transcriptome levels, enabling reproducible and regulatory-grade manufacturing.

Nevertheless, iPSC-based NK cells may differ phenotypically from their primary counterparts. Studies have reported altered receptor repertoires and differences in migratory or cytotoxic behavior. Therefore, platform optimization must include functional benchmarking in physiologically relevant models, particularly in solid tumors with dense stroma and hypoxia.

### 6.4. From Universal Cells to Contextual Precision

While universal allogeneic NK cell products remain attractive, a growing body of evidence suggests that immune evasion is not one-size-fits-all. Tumors differ significantly in their HLA landscape, NK ligand expression, and microenvironmental suppression profiles. For example, MHC class I-deficient tumors, such as microsatellite-unstable colorectal cancer, may be ideal candidates for HLA-deleted NK cells, whereas tumors with strong checkpoint expression may require concurrent disruption of TIGIT or PD-1 pathways [68,69,70].

Emerging efforts now combine patient-level multi-omics data (e.g., HLA typing, tumor RNA-Seq, single-cell immune profiling) with AI-guided modeling to predict optimal gene editing strategies. Tools such as CRISPR-AI, DeepCRISPR, and immunogenomic classifiers are being trained on large public datasets (e.g., TCGA, GTEx) to identify immunological vulnerabilities specific to tumor subtypes or patient groups [71,72].

The long-term vision is a closed-loop design pipeline in which tumor and host features are rapidly profiled, optimal NK edits are selected in silico, and cells are manufactured from standardized iPSC banks—delivering precision at the population scale. The translation of these editing strategies into personalized, context-adaptive NK cell therapies is being accelerated by the emergence of new computational and cellular tools.

### 6.5. AI-Guided Rational Design and Predictive Optimization in CAR-NK Therapy

Artificial intelligence (AI) and machine learning (ML) technologies are increasingly being applied to the development and optimization of CAR-NK cell therapies. By integrating high-throughput data, predictive modeling, and structural analytics, AI systems support rational CAR design with improved therapeutic precision.

Recent perspectives in precision oncology have emphasized the regulatory and translational challenges of integrating AI-guided strategies in CAR design, highlighting their potential to predict immune cell behavior and therapeutic efficacy [73]. Notably, AI-based platforms such as NetMHCpan, DeepCRISPR, and DeepImmuno further enable rational antigen design by predicting MHC binding, optimizing sgRNA specificity, and modeling T-cell immunogenicity, respectively [71,74,75].

Advances in mRNA delivery technologies have also benefited from AI tools that enable high-throughput optimization of CAR construct encoding, enabling more flexible and rapid design of NK-based therapies [76].

In the context of CAR expression optimization, an AI-enabled RNA engineering platform known as AGILE was used to fine-tune mRNA structures and lipid nanoparticle delivery systems for CAR immunotherapy, facilitating more efficient intracellular expression [77]. Additionally, in vivo CAR-NK production strategies now integrate AI models to streamline construct screening and reduce reliance on ex vivo processing, thereby accelerating clinical readiness [78]. Collectively, these AI-based innovations are transforming CAR-NK engineering by improving antigen specificity, enhancing cell persistence, and reducing off-target effects.

### 6.6. Practical and Regulatory Challenges

Despite exciting progress, several translational challenges remain. Multiplexed cells require robust QC systems that can detect structural variants, off-target mutations, and epigenetic drift. Moreover, regulatory authorities increasingly require long-term genotoxicity and tumorigenicity data, especially when novel gene editing tools (e.g., base editors, prime editors) are involved [79,80,81].

Additionally, scaling iPSC-derived NK cell manufacturing demands rigorous process control, validated differentiation protocols, and access to GMP-grade reagents and delivery platforms. Investment in infrastructure and regulatory science will be just as critical as scientific innovation in shaping the success of next-generation NK cell therapies [56,82].

## 7. Conclusions and Future Perspectives

Allogeneic NK cell therapy represents one of the most promising frontiers in cell-based immunotherapy. Its ability to combine rapid cytotoxicity with low risk of GVHD makes it uniquely suited for “off-the-shelf” treatment models. However, despite this potential, its full clinical realization remains incomplete—not due to a lack of innovation, but because much of that innovation has been fragmented. Numerous gene editing strategies have been proposed to address individual barriers such as T-cell rejection, NK fratricide, and phagocytic clearance, yet a cohesive framework that integrates these strategies into durable, scalable, and context-aware therapeutic platforms is still lacking.

One clear insight from recent preclinical and early clinical efforts is that immune evasion alone is insufficient. Therapeutic NK cells must do more than simply evade host immune rejection—they must persist, adapt, and maintain functional integrity within a dynamic, immunosuppressive, and often hostile tumor microenvironment. Achieving this requires a deeper reengineering of NK cell biology, moving beyond surface-level immunogenicity to fundamentally alter how NK cells respond, endure, and function.

This next phase of CAR-NK development will hinge on systemic integration, not piecemeal optimization. Multiplex editing should be approached not as an additive strategy, but as a process of immunological balancing—where each genetic modification is informed by the presence or absence of others. CAR insertion, immune checkpoint regulation, and metabolic reprogramming must be co-designed to avoid functional antagonism. In this context, the intersection of gene editing technologies, artificial intelligence, bioinformatics, and iPSC-based manufacturing opens unprecedented possibilities to create NK therapies that are both universal and adaptable to patient-specific tumor environments.

Recent progress in genome editing is redefining the therapeutic potential of CAR-NK cells. CRISPR/Cas9 technologies enable the precise deletion of immune checkpoint regulators, such as PD-1 and NKG2A, thereby improving NK cells’ cytotoxicity and persistence in the tumor microenvironment (TME) [83]. In addition, virus-free knock-in techniques now facilitate safer and more efficient integration of CAR constructs into NK-specific loci like KLRC1, improving antitumor specificity and minimizing off-target risks [84]. Beyond CRISPR, base editing approaches offer the ability to perform multiplexed, high-fidelity modifications without introducing double-stranded breaks, expanding the scope for optimizing NK cells’ function and safety [85]. These tools are expected to play a pivotal role in developing universal, off-the-shelf CAR-NK therapies with minimized immunogenicity and enhanced resilience in solid tumor environments.

However, this ambition must be tempered with caution. Each additional layer of engineering complexity, from multiplex editing to context-sensitive targeting, requires rigorous safety validation, ethical foresight, and regulatory clarity. Developers must resist viewing each immune barrier as an isolated problem and instead understand immune rejection, exhaustion, and dysfunction as interconnected manifestations of a broader immunological system.

Ultimately, the question is no longer whether allogeneic NK cell therapy *can* work—it already does, at least in part. The more pressing question is whether we can build the scientific, technical, and infrastructural ecosystem required to make it reliable, programmable, and precise. This review aimed not only to catalog current strategies but to emphasize the need for scientific synthesis. While gene editing techniques for allogeneic CAR-NK therapy are expanding rapidly, not all of them are equally ready for clinical translation. We argue that minimal-edit configurations, such as HLA-A/B deletion with HLA-C retention, or CD47 overexpression alone, may represent practical intermediate solutions before moving toward fully multiplexed systems. Furthermore, AI-guided design strategies must be grounded in real-world immune variability, supported by receptor profiling and validated using dynamic patient-derived data.

The goal is not simply maximal editing, but biologically informed and context-aware engineering. A functional CAR-NK cell is not the one with the most genetic modifications, but the one most capable of maintaining resilience and clinical performance.

While artificial intelligence offers promising avenues for optimizing CAR-NK design, it is not without critical limitations. One central challenge lies in data dependency. Most current models rely on high-quality, large-scale datasets to accurately infer relationships between immune features and therapeutic outcomes. However, such datasets are often limited, heterogeneous, or biased, particularly in NK cell research, where single-cell, time-resolved functional data remain scarce [86].

Moreover, AI models often prioritize predictive performance over biological interpretability. Black-box architectures, such as deep neural networks, may produce accurate outputs without revealing the biological rationale behind their decisions—raising issues in clinical transparency and regulatory validation [87]. Another challenge is generalizability: models trained on specific cell lines or animal models may not translate effectively to human applications, especially in diverse patient populations or solid tumor settings [88].

Finally, AI-driven insights must be integrated with experimental validation pipelines to close the loop between computation and wet-lab biology. Without this feedback, model predictions risk drifting from biological relevance. Thus, the responsible integration of AI into CAR-NK development demands not only technical sophistication but also careful attention to dataset quality, model transparency, and iterative biological validation.

In addition to enhancing therapeutic efficacy, the clinical translation of CAR-NK therapies depends heavily on their scalable and robust manufacturing. Unlike autologous CAR-T therapies, CAR-NK cell production faces unique challenges such as donor variability, limited in vitro expansion capacity, and sensitivity to cryopreservation. To address these limitations, closed bioreactor systems and feeder-free platforms using cytokine cocktails like IL-15 and IL-21 have been shown to support consistent NK cell proliferation and functional cytotoxicity [89]. Furthermore, induced pluripotent stem cell (iPSC)-derived NK cells offer a renewable and homogeneous source for CAR engineering, allowing for reproducible batch production with consistent phenotype and function [90]. The integration of automated manufacturing systems has also facilitated the GMP-compliant expansion of CAR-NK cells for clinical-grade applications [91]. These advances collectively support the feasibility of transitioning CAR-NK therapy to an off-the-shelf, scalable immunotherapy platform [92,93].

In doing so, we hope to encourage a shift from reactive design to rational construction of NK cells—not merely as products of immunology, but as tools of engineered resilience.

## Figures and Tables

**Table 1:** Summary of gene editing strategies for immune evasion in allogeneic NK cells.

Editing Target(s)	Immune Barrier(s) Addressed	Mechanism(s)	Key Risks or Limitations	Reference
B2M KO + HLA-E OE	T-cell rejection + NK fratricide	Removes classical HLA, restores inhibition via HLA-E	Multiplex editing complexity; off-target effects	[17]
HLA-A/B KO + HLA-C retention	T-cell rejection + NK tolerance	Minimizes TCR recognition while preserving NK KIR signals	HLA diversity may limit universality	[34]
CD47 OE	Macrophage clearance	Suppresses SIRPα-mediated phagocytosis	Potential tumor immune escape mimicry	[35]
HLA-E OE	NK cell inhibition (via NKG2A)	Engages NKG2A to inhibit NK activation	Not effective in NKG2C-dominant NKs	[36]
KIR editing	NK autoregulation/activation tuning	Removes inhibitory or adds activating KIRs	Loss of tolerance; tissue toxicity	[37]
CISH KO	IL-15 pathway suppression	Boosts IL-15 sensitivity and persistence	Cytokine overstimulation risk	[38]
FOXO1 KO	Transcriptional exhaustion	Promotes T-bet/Eomes expression, memory traits	Possible disruption of homeostasis	[39]
IL-1R8 KO	Infiltration + activation	Enhances chemokine response and IFN-γ secretion	Auto-inflammation potential	[40]

**Table 2:** Intrinsic genetic targets for enhancing NK cells’ persistence and effector function.

Target Gene	Biological Role	Editing Strategy	Expected Effect(s)	Preclinical Evidence	Risks
CISH	IL-15 signaling brake	Knockout	Boosts expansion, persistence, and granule release	[38]	Overstimulation, potential bystander toxicity
FOXO1	Transcriptional effector suppression	Knockout or suppression	Enhances effector programming (T-bet, Eomes)	[39]	Disruption of homeostasis, survival defects
IL-1R8	Suppressor of IL-1R signaling and chemotaxis	Knockout	Improves tumor infiltration and IFN-γ	[40]	Autoimmune-like inflammation
TIGIT	Checkpoint receptor limiting cytotoxicity	Knockout or antibody blockade	Restores NK activation and tumor killing	[54]	Loss of peripheral tolerance

**Table 3:** Ongoing clinical trials employing gene-edited allogeneic NK cells.

Developer/Trial ID	Cell Source	Gene Editing Targets	CAR Antigen	Indication	Phase	Outcomes/Status
Fate Therapeutics/NCT04555811	iPSC	B2M KO, HLA-E OE, CD38 KO	CD19	Non-Hodgkin lymphoma	Phase 1	ORR: 38%, CRS: 0%, mild cytopenia
Nkarta/NCT05020678	Peripheral blood NK	None (natural KIR mismatch)	CD19	B-ALL, CLL, NHL	Phase 1	Ongoing
MD Anderson/NCT03056339	Cord blood	IL-15 OE, iC9 safety switch	CD19	B-cell malignancies	Phase 1/2	Recruiting
Wugen/NCT05470140	Donor-derived NK	None yet	None	Acute myeloid leukemia	Phase 1	Recruiting

## Data Availability

Not applicable.
